# Timing of influenza epidemics and vaccines in the American tropics, 2002–2008, 2011–2014

**DOI:** 10.1111/irv.12371

**Published:** 2016-02-08

**Authors:** Lizette Olga Durand, Po‐Yung Cheng, Rakhee Palekar, Wilfrido Clara, Jorge Jara, Mauricio Cerpa, Nathalie El Omeiri, Alba Maria Ropero‐Alvarez, Juliana Barbosa Ramirez, Jenny Lara Araya, Belsy Acosta, Alfredo Bruno, Celina Calderon de Lozano, Leticia del Carmen Castillo Signor, Maria Luisa Matute, Sandra Jackson‐Betty, Kam Suan Mung, José Alberto Díaz‐Quiñonez, Irma López‐Martinez, Angel Balmaseda, Brechla Morneo Arévalo, Cynthia Vazquez, Victoria Gutierrez, Rebecca Garten, Marc‐Alain Widdowson, Eduardo Azziz‐Baumgartner

**Affiliations:** ^1^ Centers for Disease Control and PreventionAtlantaGAUSA; ^2^ Pan American Health OrganizationWashingtonDCUSA; ^3^ Universidad del Valle de GuatemalaGuatemala CityGuatemala; ^4^ Centro Nacional de InfluenzaLaboratorio de VirologíaInstituto Nacional De SaludBogotáColombia; ^5^ Centro Nacional de InfluenzaCentro Nacional de Referencia de VirologíaInstituto Costarricense de Investigación y Enseñanza en Nutrición y SaludSan JoseCosta Rica; ^6^ Centro Nacional de InfluenzaDepartamento de VirologíaInstituto de Medicina Tropical ‟Pedro KouríˮHavanaCuba; ^7^ El Centro de Referencia Nacional de Influenza y Otros Virus Respiratorios Insituto Nacional de Investigacion en Salud Publica (INSPI)QuitoEcuador; ^8^ Unidad de Vigilancia Laboratorial Dr. Max BlochCentro Nacional de InfluenzaSan SalvadorEl Salvador; ^9^ Centro Nacional de InfluenzaLaboratorio Nacional de SaludCiudad de GuatemalaGuatemala CityGuatemala; ^10^ Departamento de Laboratorio Nacional de Vigilancia de la SaludTegucigalpaHonduras; ^11^ National Influenza Centre‐JamaicaDepartment of MicrobiologyUniversity of West IndiesKingstonJamaica; ^12^ PAHO/WHO JamaicaNational Influenza Centre‐JamaicaDepartment of MicrobiologyUniversity of West IndiesKingstonJamaica; ^13^ National Influenza CentreInstituto de Diagnóstico y Referencia Epidemiológicos (InDRE)Ministry of HealthMexico CityMexico; ^14^ Laboratorio Nacional de VirologíaCentro Nacional de Diagnóstico y ReferenciaMinistry of HealthManaguaNicaragua; ^15^ Depto. de Investigación en Virología Instituto Conmemorativo GorgasPanamá CityPanamá; ^16^ Laboratorio Central de Salud PúblicaAsunciónParaguay; ^17^ Instituto Nacional de SaludLimaPeru

**Keywords:** Influenza, surveillence, vaccine

## Abstract

**Background:**

Influenza‐associated illness results in increased morbidity and mortality in the Americas. These effects can be mitigated with an appropriately chosen and timed influenza vaccination campaign. To provide guidance in choosing the most suitable vaccine formulation and timing of administration, it is necessary to understand the timing of influenza seasonal epidemics.

**Objectives:**

Our main objective was to determine whether influenza occurs in seasonal patterns in the American tropics and when these patterns occurred.

**Methods:**

Publicly available, monthly seasonal influenza data from the Pan American Health Organization and WHO, from countries in the American tropics, were obtained during 2002–2008 and 2011–2014 (excluding unseasonal pandemic activity during 2009–2010). For each country, we calculated the monthly proportion of samples that tested positive for influenza. We applied the monthly proportion data to a logistic regression model for each country.

**Results:**

We analyzed 2002–2008 and 2011–2014 influenza surveillance data from the American tropics and identified 13 (81%) of 16 countries with influenza epidemics that, on average, started during May and lasted 4 months.

**Conclusions:**

The majority of countries in the American tropics have seasonal epidemics that start in May. Officials in these countries should consider the impact of vaccinating persons during April with the Southern Hemisphere formulation.

## Introduction

Each year, approximately 40,880–160,270 persons die as a result of influenza‐associated illness in the Americas.^1^ Well‐timed influenza vaccines can lessen those effects.^2^ Understanding the timing of seasonal influenza epidemics can aid public health officials in determining when to vaccinate and with what formulation.

Thirty‐five of 43 countries in the Americas administer influenza vaccines, and these countries try to time their vaccination campaigns in anticipation of annual epidemics.^3^ Although timing of influenza epidemics is well described in temperate countries,^4^ those epidemics have been historically difficult to describe in the American tropics (i.e., between the Tropics of Capricorn and Cancer) because of limited availability of surveillance data.^5^, ^6^, ^7^, ^8^

During the previous decade, however, surveillance for influenza throughout the American tropics has substantially improved. Subregions such as Central America have increased the number of respiratory samples reported to the World Health Organizations (WHO) by 10‐fold.^9^ These gains have facilitated the mapping of epidemic activity in the tropics.^10^ We analyzed laboratory‐confirmed influenza data through a binomial model to describe the timing of epidemics in the American tropics. Our findings were used to make recommendations about vaccination timing and formulation.

## The study

We sought >3 years of monthly seasonal influenza data collected from countries in the American tropics during 2002–8 and 2011–2014 (excluding unseasonal pandemic activity during 2009–2010). We obtained publicly available data from the Pan American Health Organization and WHO 11, 12 where countries throughout the Americas report influenza laboratory results from respiratory specimens collected through routine clinical practice, outbreak investigations, research studies, program evaluations, and influenza‐like illness and severe acute respiratory infection surveillance.^13^ We obtained similar data from tropical Mexico (i.e., south of the Tropic of Cancer) directly from Mexico's National Influenza Centre.

We used these data to determine the expected timing of influenza activity during a typical calendar year and to ascertain the optimal month to vaccinate. For each country, we calculated the monthly proportion of samples that tested positive for influenza. This parameter served as our proxy for monthly influenza activity. We applied the monthly proportion data to a logistic regression model^1^ for each country, assuming response under a binomial distribution by using SAS^®^ PROC GENMOD (SAS Institute, Incorporated, Cary, North Carolina). The outcome was monthly influenza activity (the proportion of respiratory samples that tested positive for influenza), and explanatory variables were year and month. Predicted influenza activity was obtained from these models. We define influenza epidemics as occurring when the predicted influenza activity exceeded the annual median for ≥2 consecutive months.^4^ Epidemics that occurred during April–September was described as a Southern Hemisphere influenza season pattern and those that occurred during October–March as a Northern Hemisphere season pattern.

PAHO member countries routinely provide a purposive sample of respiratory specimens and viral isolates to CDC, their regional WHO Collaborating Centre, for antigenic characterization and oseltamivir resistance testing. We obtained antigenic strain characterization data from the Centers for Disease Control and Prevention (CDC). We determined the most common circulating strain in each country, per year and compared these data to the strains representing each type and subtype in the Northern and Southern Hemisphere influenza vaccines for the corresponding year. Finally, we assessed the proportion of years the most commonly identified antigenic strain characterization was represented in the concurrent Northern and Southern Hemisphere vaccine formulations.

**Figure 1:**
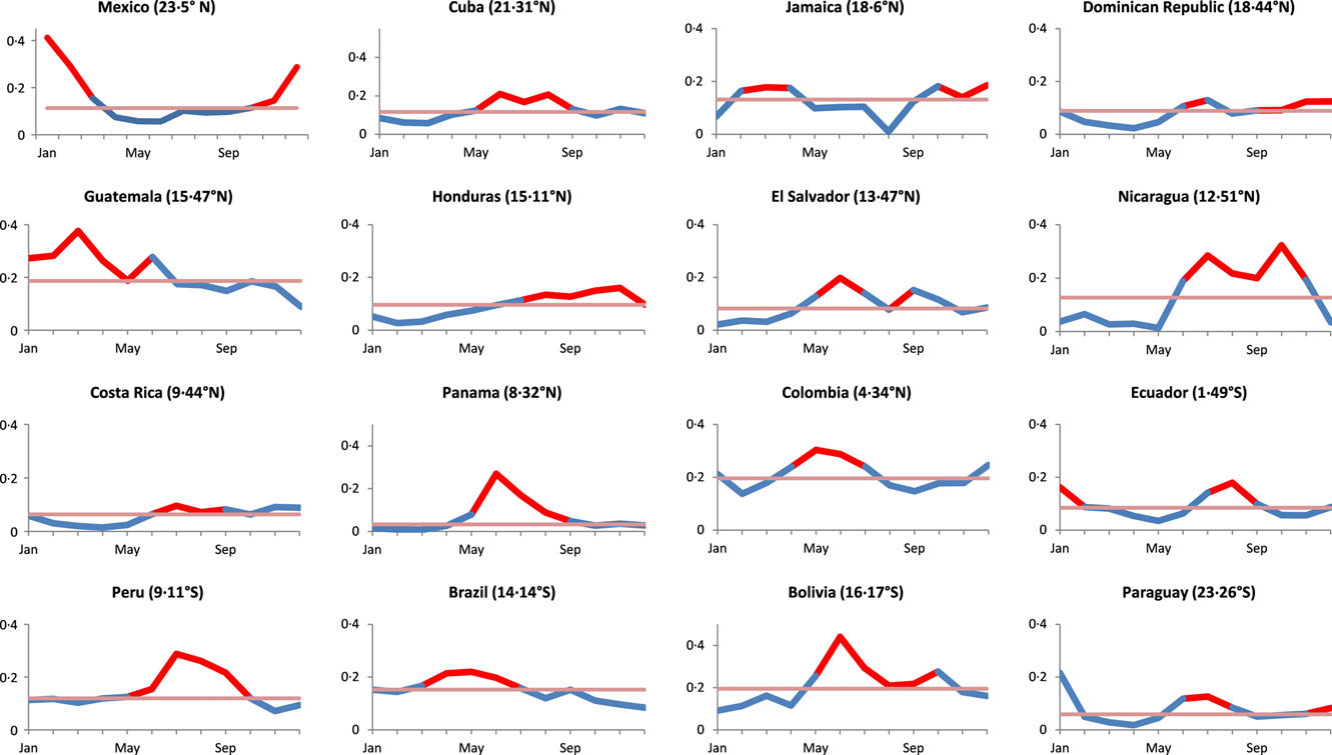
Modeled binominal yearly predicted influenza activity in the American tropics*^†‡^. *Y‐axis indicates predicted proportion of respiratory samples that tested positive for influenza. ^†^Red portion of date line indicates the epidemic period. Light red line is the median. ^‡^Study‐years for each country are as follows: Bolivia, Cuba, Dominican Republic, Ecuador, and Jamaica 2011–2014; Mexico (tropic region) 2011–2014; Brazil and Costa Rica 2003–2008 and 2011–2014; Colombia 2002–2007 and 2011–2014; Guatemala 2002, 2006–2008, and 2011–2014; El Salvador 2005–2008 and 2011–2014; Honduras, Nicaragua, and Panama 2008 and 2011–2014; Paraguay 2003–2005 and 2011–2014; and Peru 2004–2008 and 2011–2014.

We identified 16 countries in the American tropics with ≥3 years of influenza surveillance data (101 years of cumulative data) (Table 1). These countries had tested 403 584 specimens, of which 15% (75 533) were positive for influenza. According to our model, the majority (*n* = 13, 81%) of countries had influenza epidemics that started approximately during May (±2 months) and lasted an average of 4 months (Figure 1). The exceptions to this pattern were Guatemala and Mexico, which had influenza epidemics primarily in a Northern Hemisphere pattern, and Jamaica, which had epidemics as early as February. With the exception of these 3 countries, April–September epidemics accounted for an average of 68% of the annual influenza activity.

**Table 1:** Influenza activity in the American tropics, 2002–2014

Country	Average samples tested/year	Years with >1 epidemic (%) years of data	Median start (95% CI) of April–September epidemics	Median length (95% CI) of April–September epidemics^a^	Influenza activity during April–September (%)^b^	Median start (95% CI) of October–March epidemics	Median length (95% CI) of October–March epidemics^a^	Influenza activity during October–March (%)^b^
Bolivia	5619	0 (0) 4	May (May–May)	6 (5–7)	66	NA	NA	34
Brazil	6404	0 (0) 10	March (Jan–Apr)	5 (3–7)	79	NA	NA	21
Colombia	4288	10 (100) 10	April (Mar–Apr)	4 (4–6)	63	December (Dec–Dec)	2 (2–4)	37
Costa Rica	2018	10 (100) 10	June (Jun–Jun)	4 (2–8)	48	November (Jun–Nov)	2 (2–2)	53
Cuba	4216	0 (0) 4	May (Apr–Jun)	5 (3–6)	55	NA	NA	45
Dominican Republic^c^	1049	4 (100) 4	June (Jun–Jun)	2 (2–8)	47	September (Jun–Nov)	4 (2–8)	53
Ecuador	10 375	4 (100) 4	July (Jul–Jul)	3 (3–3)	52	December (Dec–Dec)	3 (2–4)	48
El Salvador^d^	1449	8 (100) 8	May (Apr–May)	3 (3–9)	78	NA	NA	22
Guatemala	1647	0 (0) 8	NA	NA	46	January (Jan–Jan)	6 (4–11)	54
Honduras	1411	0 (0) 5	July (May–Aug)	6 (4–8)	53	NA	NA	47
Jamaica^e^	466	4 (100) 4	NA	NA	30	October (Sep–Oct)	3 (2–11)	60
Mexico^f^	3270	0 (0) 4	NA	NA	25	November (Nov–Dec)	5 (4–7)	75
Nicaragua	4498	0 (0) 5	June (Jun–Jun)	6 (6–6)	51	NA	NA	49
Panama	1739	0 (0) 5	May (Apr–May)	5 (5–9)	89	NA	NA	11
Paraguay	7532	7 (100) 7	June (Jun–Jun)	3 (3–8)	72	November (Nov–Dec)	3 (2–8)	28
Peru	3277	0 (0) 9	May (Jan–Jun)	6 (4–10)	76	NA	NA	24
Total	3704	47 (47) 101	May (Apr–Jun)	4 (3–7)	58	November (Oct–Dec)	4 (2–7)	41

NA = not applicable because there was no consistently identified epidemic during that time of the year.

a Not every country had a secondary influenza epidemic season, every year. To calculate the length of this season, we only accounted for those years that had a secondary influenza season lasting at least 2 months.

b Influenza activity from April–September and October–March is based on the proportion of samples tested during that time period.

c Since the September epidemic period extended through December, it was assigned to the October–March period.

d The modeled El Salvador epidemic period dipped just below the median in August and then continued for 2 months. The primary season was extended to include the additional months.

e Jamaica had a short 3‐month season during February–April.

f We only analyzed Mexico data south of the Tropic of Cancer.

Thirty‐eight percent (*n* = 5) of the 13 countries with epidemics that occur approximately during April–September (±2 months) also had sporadic influenza activity that started during November (±2 months) and lasted an average of 3 months (Figure 1). October–March epidemics occurred in 39 (39%) of the 101 study‐years.

We analyzed 78 country‐years of antigenic characterization data collected during 2006–2008, 2011–2014. The most commonly identified antigenic characterization matched a component strain of the concurrent Southern Hemisphere vaccine formulation during 51 (65%) of 78 study‐years, and of the Northern Hemisphere vaccine formulation during 42 (54%) of the study‐years (Table 2), but this difference was not statistically significant.

**Table 2:** Predominant antigenic characterization of influenza strain compared with vaccine formulation during 2002–2008, 2011–2014

Country	Years of available data	Years predominant strain represented in	
		Southern Hemisphere vaccine, %	Northern Hemisphere vaccine, %
Bolivia	2	100	50
Brazil	12	75	50
Colombia	1	100	0
Costa Rica	8	50	50
Dominican Republic	2	100	100
Ecuador	1	100	100
El Salvador	9	56	44
Guatemala	7	57	43
Honduras	7	43	57
Jamaica	1	100	100
Nicaragua	7	57	57
Panama	9	44	44
Paraguay	9	89	67
Peru	3	100	67
Total	78	65	54

The predominant strain each year could be included in both Northern and Southern Hemisphere formulation vaccine recommendations.

**Figure 2:**
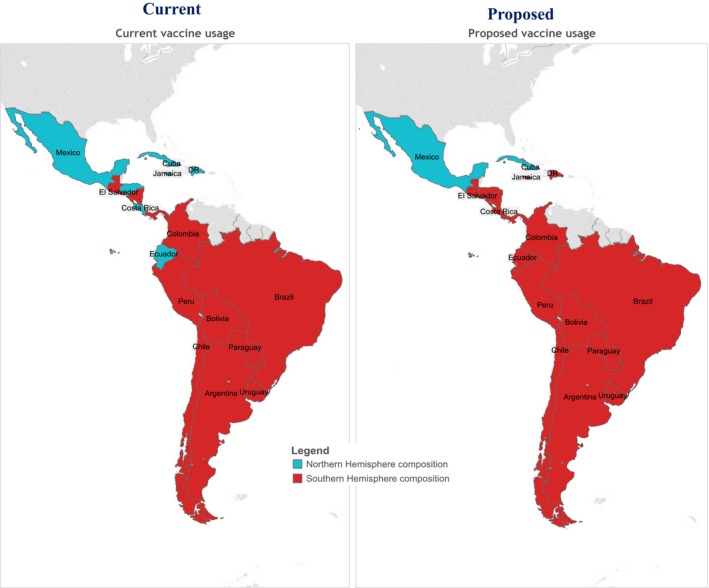
Current and proposed influenza vaccine formulation type for the American tropics.

Our analysis of laboratory data from the American tropics indicates that all 16 study countries had ≥1 annual influenza epidemic that lasted ~4 months. With the exception of Guatemala, Jamaica, and tropical Mexico, annual epidemics in the American tropics typically occurred during April–September. Indeed, influenza activity during April–September accounted for approximately two‐thirds (2/3) of the annual influenza activity. Although secondary epidemics that lasted approximately for 3 months occurred during October–March, these were of a limited magnitude when compared to the primary May–September epidemics. October–March influenza activity typically accounted for 41% of influenza activity. With the exception of Guatemala, Jamaica, and tropical Mexico, countries in the American tropics seem justified in vaccinating against influenza during the April *Vaccination Week of the Americas* 3, 14 in anticipation of a primary influenza risk period during April–September (Figure 2).

The influenza strains characterized during the primary epidemics frequently matched the components recommended for the Southern Hemisphere influenza vaccine. The selection of components for the Southern Hemisphere vaccine occurs during September, when experts examine surveillance data and determine which strains are anticipated during austral winter.^15^ Southern Hemisphere vaccines typically become available during late March–early April and have the most up‐to‐date formulations available to protect against late April–September influenza epidemic activity. With the exception of Guatemala, Jamaica, and tropical Mexico, countries in the American tropics should consider the potential benefits of vaccinating with the Southern Hemisphere vaccine formulations during early April to provide protection against anticipated influenza activity during April–September. Even countries with two annual influenza epidemics in some or all of their provinces (e.g., Brazil and Ecuador) 16 should consider the effect of increasing vaccination coverage among target populations during the main influenza epidemic in April–September before investing in a second vaccination campaign during October–November. Tropical Mexico and Jamaica already vaccinate with Northern Hemisphere formulation influenza vaccines, and Guatemala public health officials might consider the potential benefits of this strategy as well.

Our study had several limitations. Sampling and diagnostic methods varied among and within countries. We only had 4 years of data from Bolivia, Jamaica, and Ecuador where the primary influenza epidemic periods were more difficult to discern. Although all 16 of our study countries are considered tropical, they differ substantially in landscape and climate. Mexico and Peru, for instance, are ecologically diverse, and previous studies have shown differences in the timing of epidemic activity between climate zones.

## Conclusion

Each year, influenza epidemics occurred during April–September in most of the American tropics. Although secondary epidemics occurred frequently, the majority of influenza cases occurred during April–September. The most up‐to‐date and representative influenza vaccine available at the start of this primary epidemic period was the Southern Hemisphere formulation. Countries in this region should explore the potential benefits of deploying Southern Hemisphere influenza vaccine campaigns during April. With the exception of Guatemala, Jamaica, and tropical Mexico, countries within the American tropics that are using Northern Hemisphere vaccine should explore the potential value of switching to the Southern Hemisphere vaccine formulations.

## Supporting information


**Table S1.** Strain information for the most prevalent circulating influenza strains in our study, per country, per year.

## Note

1
$\begin{array}{cc} & \log\left(\frac{\text{Proportion of samples testing positve for influenza}}{1-\text{proportion of samples testing positive for influenza}}\right)\\ & \;=\text{year}+\text{month} \end{array}$
