# Supplementary material for: Timing of influenza epidemics and vaccines in the American tropics, 2002–2008, 2011–2014
Source: Influenza Other Respir Viruses. 2016 Feb 8;10(3):170–5. doi: 10.1111/irv.12371 (PMC4814866; doi:10.1111/irv.12371)
Supplement: Supplementary file 1 — Table S1. Strain information for the most prevalent circulating influenza strains in our study, per country, per year. [file IRV-10-170-s001.docx]

| **Year** | **Southern Vaccine Formulation** | **Northern Vaccine Formulation** | **Country** | **Circulating Influenza Strain** |
| --- | --- | --- | --- | --- |
| 2001  /2000-2001 | A/Moscow/10/99 (H3N2)-like virus^1^  A/New Caledonia/20/99 (H1N1)-like virus  B/Sichuan/379/99-like virus | A/New Caledonia/20/99(H1N1)-like virus  A/Moscow/10/99(H3N2)-like virus  B/Beijing/184/93-like virus | Brazil | A/PANAMA/2007/99-LIKE (H3N2)^*^ |
| 2002  /2001-2002 | A/New Caledonia/20/99(H1N1)-like virus  A/Moscow/10/99(H3N2)-like virus  B/Sichuan/379/99-like virus | A/New Caledonia/20/99(H1N1)-like virus  A/Moscow/10/99(H3N2)-like virus  B/Sichuan/379/99-like virus | Brazil Panama | B/SHANDONG/7/97-LIKE (VIC) B/SHANDONG/7/97-LIKE (VIC) |
| 2003  /2002-2003 | A/New Caledonia/20/99(H1N1)-like virus  A/Moscow/10/99(H3N2)-like virus  B/Hong Kong/330/2001-like virus^2^ | A/New Caledonia/20/99(H1N1)-like virus  A/Moscow/10/99(H3N2)-like virus B/Hong Kong/330/2001-like virus | Brazil El Salvador Nicaragua Paraguay | A/NEW CALEDONIA/20/99-LIKE (H1) A/FUJIAN/411/2002-LIKE(H3) A/FUJIAN/411/2002-LIKE(H3)^*^ B/SHANDONG/7/97-LIKE (VIC)^*^ |
| 2004  /2003-2004 | A/New Caledonia/20/99(H1N1) -like virus  A/Fujian/411/2002(H3N2) - like virus  B/Hong Kong/330/2001-like virus | A/New Caledonia/20/99(H1N1)-like virus  A/Moscow/10/99(H3N2)-like virus  B/Hong Kong/330/2001-like virus^2^ | Brazil El Salvador Panama Paraguay | A/FUJIAN/411/2002-LIKE(H3) A/FUJIAN/411/2002-LIKE(H3) B/SHANGHAI/361/2002-LIKE (YAM) A/FUJIAN/411/2002-LIKE(H3) |
| 2005  /2004-2005 | A/New Caledonia/20/99(H1N1)-like virus  A/Wellington/1/2004(H3N2)-like virus  B/Shanghai/361/2002-like virus | A/New Caledonia/20/99(H1N1)-like virus  A/Fujian/411/2002(H3N2)-like virus  B/Shanghai/361/2002-like virus | Brazil Costa Rica El Salvador Honduras Panama Paraguay | A/CALIFORNIA/07/2004-LIKE (H3N2) A/CALIFORNIA/07/2004-LIKE (H3N2) LOW A/CALIFORNIA/07/2004-LIKE (H3N2) A/CALIFORNIA/07/2004-LIKE (H3N2) A/CALIFORNIA/07/2004-LIKE (H3N2) A/CALIFORNIA/07/2004-LIKE (H3N2) |
| 2006  /2005-2006 | A/New Caledonia/20/99(H1N1)-like virus  A/California/7/2004(H3N2)-like virus  B/Malaysia/2506/2004-like virus | A/New Caledonia/20/99(H1N1)-like virus  A/California/7/2004(H3N2)-like virus  B/Shanghai/361/2002-like virus | Brazil Costa Rica El Salvador Guatemala Honduras Paraguay | A/NEW CALEDONIA/20/99-LIKE (H1) A/WISCONSIN/67/2005-LIKE (H3N2) A/CALIFORNIA/07/2004-LIKE (H3N2) A/WISCONSIN/67/2005-LIKE (H3N2) A/WISCONSIN/67/2005-LIKE (H3N2) LOW^*^ A/NEW CALEDONIA/20/99-LIKE (H1) |
| 2007  /2006-2007 | A/New Caledonia/20/99(H1N1)-like virus  A/Wisconsin/67/2005(H3N2)-like virus  B/Malaysia/2506/2004-like virus | A/New Caledonia/20/99(H1N1)-like virus  A/Wisconsin/67/2005 (H3N2)-like virus  B/Malaysia/2506/2004-like virus | Brazil Costa Rica El Salvador Guatemala Honduras Nicaragua Panama | A/WISCONSIN/67/2005-LIKE (H3N2) LOW A/BRISBANE/10/2007-LIKE (H3N2) A/BRISBANE/10/2007-LIKE (H3N2) A/BRISBANE/10/2007-LIKE (H3N2) A/BRISBANE/10/2007-LIKE (H3N2)^*^ A/BRISBANE/10/2007-LIKE (H3N2) A/BRISBANE/10/2007-LIKE (H3N2) |
| 2008  /2007-2008 | A/Solomon Islands/3/2006 (H1N1)-like virus  A/Brisbane/10/2007 (H3N2)-like virus  B/Florida/4/2006-like virus | A/Solomon Islands/3/2006 (H1N1)-like virus  A/Wisconsin/67/2005 (H3N2)-like virus  B/Malaysia/2506/2004-like virus | Brazil Costa Rica El Salvador Guatemala Honduras Nicaragua Panama Paraguay | A/BRISBANE/59/2007-LIKE (H1N1) A/BRISBANE/59/2007-LIKE (H1N1) A/BRISBANE/59/2007-LIKE (H1N1) A/BRISBANE/10/2007-LIKE (H3N2) A/BRISBANE/59/2007-LIKE (H1N1) A/BRISBANE/59/2007-LIKE (H1N1) A/BRISBANE/59/2007-LIKE (H1N1) B/FLORIDA/04/2006-LIKE (YAM) |
| 2011  /2010-2011 | A/California/7/2009 (H1N1)-like virus  A/Perth/16/2009 (H3N2)-like virus  B/Brisbane/60/2008-like virus | A/California/7/2009 (H1N1)-like virus  A/Perth/16/2009 (H3N2)-like virus  B/Brisbane/60/2008-like virus | Brazil Costa Rica El Salvador Guatemala Honduras Nicaragua Panama Paraguay | A/PERTH/16/2009-LIKE (H3N2) A/PERTH/16/2009-LIKE (H3N2) A/PERTH/16/2009-LIKE (H3N2) A/PERTH/16/2009-LIKE (H3N2) B/BRISBANE/60/2008-LIKE A/CALIFORNIA/07/2009-LIKE (H1N1)pdm09 A/CALIFORNIA/07/2009-LIKE (H1N1)pdm09 A/PERTH/16/2009-LIKE (H3N2) |
| 2012  /2011-2012 | A/California/7/2009 (H1N1)pdm09-like virus  A/Perth/16/2009 (H3N2)-like virus  B/Brisbane/60/2008-like virus | A/California/7/2009 (H1N1)-like virus  A/Perth/16/2009 (H3N2)-like virus  B/Brisbane/60/2008-like virus | Brazil Costa Rica El Salvador Guatemala Honduras Nicaragua Panama Paraguay Peru | A/CALIFORNIA/07/2009-LIKE (H1N1)pdm09 B/BRISBANE/60/2008-LIKE A/CALIFORNIA/07/2009-LIKE (H1N1)pdm09 A/CALIFORNIA/07/2009-LIKE (H1N1)pdm09 A/CALIFORNIA/07/2009-LIKE (H1N1)pdm09 B/BRISBANE/60/2008-LIKE A/CALIFORNIA/07/2009-LIKE (H1N1)pdm09 A/CALIFORNIA/07/2009-LIKE (H1N1)pdm09 A/CALIFORNIA/07/2009-LIKE (H1N1)pdm09 |
| 2013  /2012-2013 | A/California/7/2009 (H1N1)pdm09-like virus  A/Victoria/361/2011 (H3N2)-like virus  B/Wisconsin/1/2010-like virus | A/California/7/2009 (H1N1)pdm09-like virus  A/Victoria/361/2011 (H3N2)-like virus  B/Wisconsin/1/2010-like virus | Bolivia Brazil Costa Rica D.R. Ecuador El Salvador Guatemala Jamaica Nicaragua Panama Paraguay Peru | A/CALIFORNIA/07/2009-LIKE (H1N1)pdm09 A/CALIFORNIA/07/2009-LIKE (H1N1)pdm09 A/CALIFORNIA/07/2009-LIKE (H1N1)pdm09 A/CALIFORNIA/07/2009-LIKE (H1N1)pdm09 A/CALIFORNIA/07/2009-LIKE (H1N1)pdm09 A/VICTORIA/361/2011-LIKE (H3N2) GP B/MASSACHUSETTS/02/2012-LIKE A/CALIFORNIA/07/2009-LIKE (H1N1)pdm09^*^ A/CALIFORNIA/07/2009-LIKE (H1N1)pdm09 A/CALIFORNIA/07/2009-LIKE (H1N1)pdm09 A/CALIFORNIA/07/2009-LIKE (H1N1)pdm09 A/CALIFORNIA/07/2009-LIKE (H1N1)pdm09 |
| 2014  /2013-2014 | A/California/7/2009 (H1N1)pdm09-like virus  A/Texas/50/2012 (H3N2)-like virus  B/Massachusetts/2/2012-like virus | A/California/7/2009 (H1N1)pdm09-like virus  A/Victoria/361/2011  B/Massachusetts/2/2012-like virus | Bolivia Brazil Colombia Costa Rica D.R. Guatemala Honduras Nicaragua Panama Paraguay Peru | A/TEXAS/50/2012-LIKE (H3N2) GP A/TEXAS/50/2012-LIKE (H3N2) GP A/TEXAS/50/2012-LIKE (H3N2) GP B/MASSACHUSETTS/02/2012-LIKE B/MASSACHUSETTS/02/2012-LIKE B/MASSACHUSETTS/02/2012-LIKE B/MASSACHUSETTS/02/2012-LIKE B/MASSACHUSETTS/02/2012-LIKE B/MASSACHUSETTS/02/2012-LIKE A/CALIFORNIA/07/2009-LIKE (H1N1)pdm09 A/TEXAS/50/2012-LIKE (H3N2) GP |
| *The strain was prevalent during the last quarter of the year, after the Northern Vaccine was rolled out, therefore, it was compared to the following years Northern Vaccine  ^1^The widely used vaccine strain is A/Panama/2007/99  ^2^A currently used vaccine strain is B/Shandong/7/97 | | | | |

Supplemental Table 1. Strain information for the most prevalent circulating influenza strains in our study, per country, per year.
